# Experimental Study on the Flexural Performance of Steel–Polyvinyl Alcohol Hybrid Fiber-Reinforced Concrete

**DOI:** 10.3390/ma17133099

**Published:** 2024-06-25

**Authors:** Jingjiang Wu, Wenjie Zhang, Juhong Han, Zheyuan Liu, Jie Liu, Yafei Huang

**Affiliations:** 1 China Construction Seventh Engineering Division Co., Ltd., Zhengzhou 450003, China; wujingjiang@cscec.com (J.W.); liujie@cscec.com (J.L.); huangyafei@cscec.com (Y.H.); 2 CCCC Second Harbour Engineering Co., Ltd., Wuhan 430040, China; 3 School of Water Conservancy and Transportation, Zhengzhou University, Zhengzhou 450001, China; 13015112337@163.com

**Keywords:** panel concrete, steel–PVA hybrid fibers, flexural performance, fractal theory

## Abstract

This paper explores the impact of steel–PVA hybrid fibers (S-PVA HF) on the flexural performance of panel concrete via three-point bending tests. Crack development in the concrete is analyzed through Digital Image Correlation (DIC) and Scanning Electron Microscope (SEM) experiments, unveiling the underlying mechanisms. The evolution of cracks in concrete is quantitatively analyzed based on fractal theory, and a predictive model for flexural strength (PMFS) is established. The results show that the S-PVA HF exhibits a synergistic effect in enhancing and toughening the concrete at multi-scale. The crack area of steel–PVA hybrid fiber concrete (S-PVA HFRC) is linearly correlated with deflection (*δ*), and it further reduces the crack development rate and crack area compared to steel fiber-reinforced concrete (SFRC). The S-PVA HF improves the proportional ultimate strength (*f*_L_) and residual flexural strength (*f*_R,j_) of concrete, and the optimal flexural performance of concrete is achieved when the steel fiber dosage is 1.0% and the PVA fiber dosage is 0.2%. The established PMFS of hybrid fiber-reinforced concrete (HFRC) can effectively predict the flexural strength of concrete.

## 1. Introduction

The concrete panel of the concrete-faced rockfill dam (CFRD) is the main anti-seepage structure of the dam body, and the safety of the panel is related to the overall stability of the dam [1,2]. Compared to other dam types, CFRD has significant advantages in terms of adaptability, safety, and economy. However, the thin-layer structure of the panels makes them prone to cracking. Improving the flexural performance of panel concrete is crucial for crack resistance and the seepage prevention of CFRD [3,4,5,6,7,8].

A three-point bending test of the concrete was performed to examine the flexural performance of the concrete specimen and to monitor the formation and propagation of cracks during the bending test as well as the specimen’s total failure. This was carried out in order to investigate the flexural performance of panel concrete [9,10]. Adding fibers, such as steel or PVA, to the concrete to improve its brittleness is one practical method to improve concrete’s flexural performance [11,12,13].

The addition of steel fibers can improve the compressive, tensile, impact, and fatigue properties and the durability of concrete [14,15,16,17,18,19,20,21,22]. The steel fibers spanning across cracks prevent rapid crack propagation, significantly enhancing the flexural performance of concrete [23,24]. Chen et al. [25] studied the effect of different steel fiber shapes and content on the bending performance of concrete. They found that the post-cracking behavior of SFRC evolved from strain softening to strain hardening by increasing the fiber content from 0 to 110 kg/m^3^. Moreover, 4D and 5D improved the bending toughness of concrete better than 3D steel fibers. Luo et al. [26] found similar conclusions in recycled coarse aggregate concrete. Gondokusumo [27] investigated the influence of high temperature on the $f_{L}$ and $f_{R,j}$ of concrete with different steel fiber volume fraction ($V_{f}$). They found that under different high-temperature conditions, as the steel fiber $V_{f}$ increased to 1.5%, the $f_{L}$ and $f_{R,j}$ of SFRC increased. When the steel fiber $V_{f}$ was constant, the $f_{u}$ of SFRC gradually decreased with increasing temperature. Xu [28] studied the effect of steel fibers on the flexural performance of rubber concrete containing ceramic waste materials. They found that compared with the reference concrete, the $f_{u}$ of SFRC with steel fiber dosages of 1.0% and 1.25% increased by 4.8% and 9.5%, respectively. In a study conducted by Tiberti [29], the impact of steel fiber dosage, shape, and orientation on the flexural performance of concrete was explored. It was observed that the *f*_L_ and $f_{R,j}$ values of SFRC increased with higher steel fiber dosage (0.38% to 0.76%). Moreover, the orientation of steel fibers played a crucial role in crack development. Overall, these studies indicate that steel fibers can greatly improve the flexural performance of concrete, although their effectiveness in inhibiting the generation and development of microcracks is relatively limited.

Polyvinyl alcohol (PVA) fibers, derived from materials such as polyvinyl alcohol, are synthetic fibers known for their hydrophilicity and strong bond with the cement matrix. When incorporated into concrete, PVA fibers effectively inhibit microcrack formation and development, resulting in improved compressive strength, splitting tensile strength, and toughness [30,31,32]. Wang [33] investigated the impact of PVA fibers on the flexural performance of rubber concrete and found a significant enhancement in toughness. PVA fiber dosage of 0.5% increased the CMOD (Crack Mouth Opening Displacement) value, achieving a threefold improvement compared to reference concrete. Jang [34] studied the effect of PVA fibers on the flexural performance of concrete after freeze–thaw cycles and observed a notable increase in *f*_u_ after 150 cycles. Li [35] studied the effect of PVA fibers on the $f_{u}$ of cement mortar and discovered that as the PVA fiber dosage (ranging from 0% to 2.0% by volume) increased, the improvement in $f_{u}$ and bending toughness gradually intensified. These studies collectively indicate that PVA fibers can enhance the ductility of concrete or cement mortar and suppress microcrack formation. Notably, the mentioned studies employed coarse aggregate particle sizes not exceeding 25 mm.

Steel fibers, by bridging cracks, prevent rapid crack propagation, while PVA fibers, with their large quantity and high strength and elastic modulus as polymer fibers, inhibit the generation and development of microcracks. Mixing steel fibers and PVA fibers can enhance the mechanical properties of concrete, such as compressive strength and tensile strength [36,37,38,39,40,41,42]. Liu [43] investigated the influence of S-PVA HF on the $f_{u}$ of concrete containing slag powder and fly ash. The study found that increasing the dosage of steel fibers and PVA fibers improved both the *f*_u_ and toughness of HFRC, with the effect of steel fibers being more significant than that of PVA fibers. Currently, there is limited research on the flexural performance of S-PVA HF in panel concrete with a maximum coarse aggregate particle size of 40 mm. To enhance the flexural performance of panel concrete, it is particularly important to study the impact of S-PVA HF on flexural performance. Analyzing the improvement of the flexural performance of concrete with S-PVA HF at different δ and conducting three-point bending tests using the European standard BS EN 14651:2005+A1:2007 [44] are crucial aspects of this research.

DIC techniques [45,46,47,48,49,50,51] are widely used in observing crack development. By analyzing and studying the displacement field distribution and characteristics of the specimen surface from loading to failure, DIC provides accurate digital information about the real morphology and location of cracks. It has been applied in concrete compressive tests, splitting tensile tests, uniaxial compression tests, and bending tests. However, there is limited research on the quantitative analysis of crack development.

To improve the flexural performance of panel concrete, this study conducted three-point bending tests on S-PVA HFRC. DIC was used to observe the generation and development of cracks on the surface of the concrete, and SEM tests were carried out to explore the toughening and crack inhibition mechanisms of S-PVA HF within the concrete. Based on fractal theory, the evolution of cracks in S-PVA HFRC was quantitatively analyzed, and a PMFS of HFRC was established.

## 2. Experimental Materials and Methods

### 2.1. Experimental Materials

**Figure 1:**
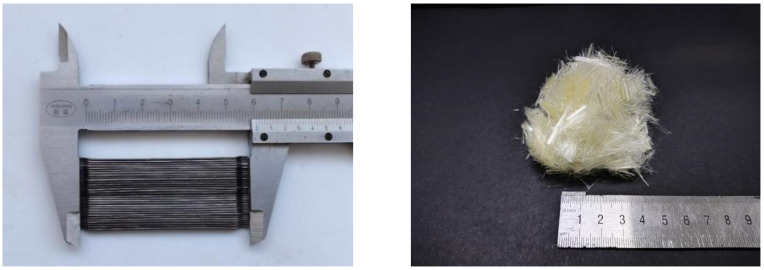
Steel fiber and PVA fiber diagram.

In this experiment, the following raw materials were used, each with their respective parameters and properties. P.O42.5 cement served as the cement type. The coarse aggregate comprised crushed stones ranging from 5 mm to 40 mm, with small stones (5 mm–20 mm) accounting for 55% and large stones (20 mm–40 mm) accounting for 45%. The fine aggregate (fineness modulus is 2.63) was obtained from natural river sand and continuous grading. Sand and gravel provided by Nanjing Convergence Stone Co., Ltd. (Nanjing, China). Three-dimensional hooked-end steel fibers with a length of 60 mm were used as the steel fibers, as depicted in Figure 1. Steel fibers utilized were supplied by Yutian Zhitai Steel Fiber Manufacturing Co., Ltd. (Tangshan, China). The PVA fibers utilized were supplied by Kuraray Co., Ltd. (Tokyo, Japan), with a diameter of 31 μm and a length of 12 mm, as shown in Table 1 and Figure 1. A high-efficiency polycarboxylate-based water-reducing agent was employed. High-quality Class I fly ash was utilized. The experiment employed tap water sourced from the local supply.

According to Chinese standard SL228-2013 [52], panel concrete should have a 28-day strength grade of at least C25 and a water–binder ratio below 0.45, and fly ash-substituting cement should be limited to 30%. In this experiment, Chinese standard DL/T5330-2015 [53] and Chinese standard JG/T472-2015 [54] were referenced. The assumed mass method was employed to prepare panel concrete (C30), where 20% of the cement was replaced by fly ash. Nine concrete batches were determined using calculated mix proportions (Table 2). The compressive strength of the concrete cubes is presented in Table 3.

### 2.2. Test Methods

#### 2.2.1. Three-Point Bending Test

**Figure 2:**
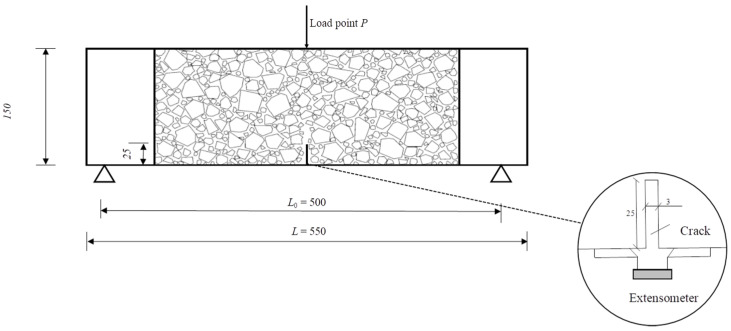
Diagram of the concrete beam specimen.

**Figure 3:**
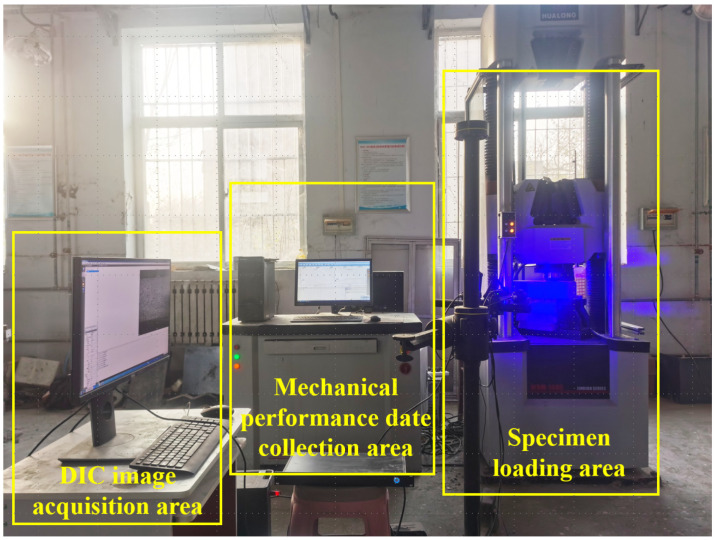
Diagram of the loading setup for the concrete.

Following the standard BS EN 14651:2005+A1:2007 [44], a total of 54 beams measuring 150 mm × 150 mm × 550 mm were cast for the three-point bending test. After 24 h, they were demolded and subsequently cured for 28 days in a standard curing chamber (RH > 95%, temperature is 20 ± 2 °C). To measure the CMOD, an extensometer was installed at the notch position, as depicted in Figure 2. The three-point bending test was performed according to the European standard, utilizing a displacement rate of 0.05 mm/min (for displacements from 0 to 0.1 mm) and 0.2 mm/min (for displacements from 0.1 to 3.5 mm). The DIC technique was utilized to capture the failure mode of the specimens during loading, as shown in Figure 3. Crack formation and extension during the three-point bending tests of concrete were observed. If any specimen did not exhibit cracking from the pre-cut notch, the test result was excluded. The $f_{u}$, $f_{L}$, and $f_{R,j}$ values of the concrete were calculated using Equations (1)–(3), respectively.

(1) $$f_{u}=\frac{3F_{u}L}{2b{h_{\mathrm{sp}}}^{2}}$$

(2) $$f_{L}=\frac{3F_{L}L}{2b{h_{\mathrm{sp}}}^{2}}$$

(3) $$f_{R,j}=\frac{3F_{j}L}{2b{h_{\mathrm{sp}}}^{2}}$$

In the equations: *L*—Support span (mm); see abbreviations for other parameters.

#### 2.2.2. Microstructure Test

**Figure 4:**
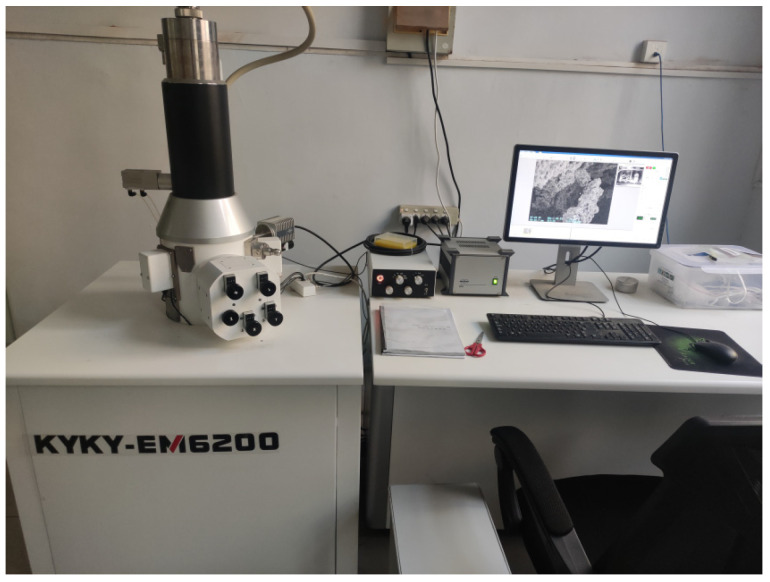
Image of KYKY-EM6200 scanning electron microscope.

The KYKY-EM6200 scanning electron microscope, depicted in Figure 4, was utilized in this experiment. It offers a magnification range of 6×–100,000×, with a secondary electron imaging resolution of 3 nm at an applied voltage of 30 kV and 10 nm at an applied voltage of 3 kV. The preparation of specimens took place after the completion of the three-point bending test.

#### 2.2.3. Fractal Dimension

Gangepain and Roques-Carms introduced the fractal dimension (*D*) measure based on box counting. This method involves calculating the minimum number of boxes required to cover the surface of an image. The size of the image is denoted as ‘L’, where ‘k’ represents the grid size and ‘*N*_r_’ represents the number of boxes needed to cover the entire image. To determine *D*, a series of *N*_r_ values is calculated by varying the grid size ‘k’. Then, a linear regression is performed on the pairs of points {log(1/*r*), log(*N*_r_)}. The slope of the regression line corresponds to the *D* value, as described in Equation (4).

(4) $$D=lim\frac{log(N_{r})}{log(1/r)}$$

In the equation, *D* represents the fractal dimension, and *N*_r_ represents the number of boxes covering the entire image.

## 3. Results and Discussion

### 3.1. Concrete Failure Process and Failure Mode

#### 3.1.1. Reference Concrete

**Figure 5:**
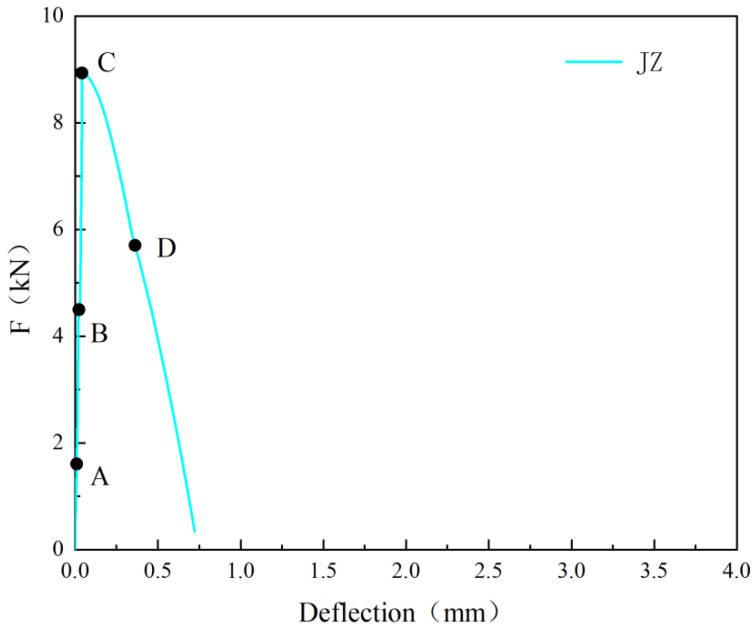
F-*δ* curve of the reference concrete.

**Figure 6:**
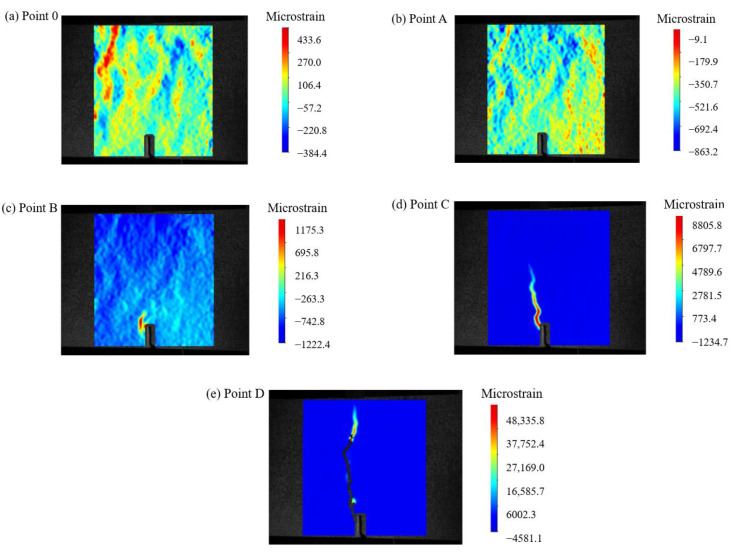
DIC measurement values at different nodes of the reference concrete.

To provide a clear description of the propagation of cracks during the test of the reference concrete-notched beams, Figure 5 shows the *F*-*δ* curve of the reference concrete, and Figure 6 presents the DIC measurement values at various stages.

From Figure 5 and Figure 6, it can be observed that during the pre-peak stage (before point C in Figure 5), no macroscopic cracks were formed. The strain distribution was uniform before point A, and stress concentration started to occur at the pre-existing crack location (point B), resulting in relatively small strain values. At this point, the *F* was 4.5 kN. As the *F* increased to 8.94 kN, the peak *F* of the reference concrete was reached, and the strain values reached their maximum. After point C, with a further increase in δ, the reference concrete rapidly developed cracks, and the *F* dropped sharply. The first crack was observed (point D), almost the entire height of the sample.

#### 3.1.2. SFRC

**Figure 7:**
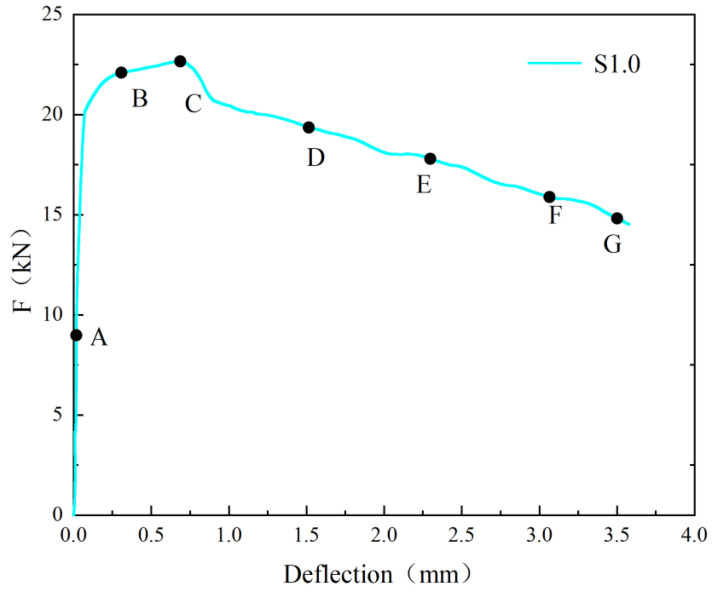
F-*δ* curve of SFRC.

**Figure 8:**
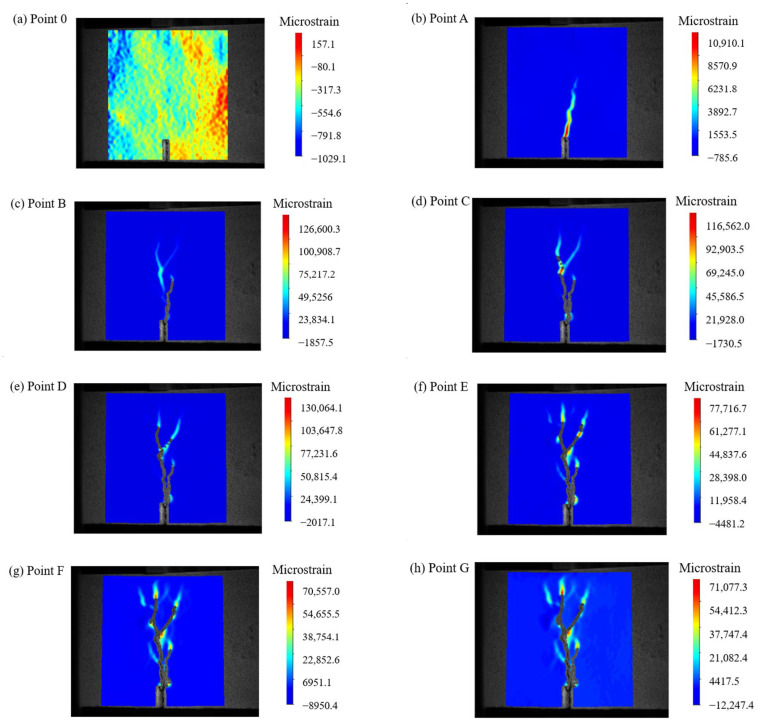
DIC Measurements at different points of SFRC.

To clearly illustrate the propagation of cracks in the SFRC during the test, Figure 7 presents the *F*-*δ* curve of S1.0, and Figure 8 shows the DIC measurement values at various stages.

Based on Figure 7 and Figure 8, it can be seen that in the pre-peak stage (before point A in Figure 7), no macroscopic cracks were visible, and the strain distribution was uniform with relatively low values. As *F* approached point A, stress concentration phenomena occurred. When *F* increased to 22.1 kN, the first crack was observed (point B). Prior to reaching peak *F* (point C), the slope of the *F*-δ curve decreased. Once *F* reached its maximum value (point C), further loading resulted in the continued expansion of the main crack, and at a *δ* of 3.5 mm (point G), the main crack nearly traversed the entire section. Due to the steel fibers, the development of cracks was affected and new crack directions were created. The width of these cracks increased with the increasing δ. The final failure occurred above the pre-existing crack, which was consistent with the findings of Banthia [55].

#### 3.1.3. S-PVA HFRC at the δ Is 3 mm

**Figure 9:**
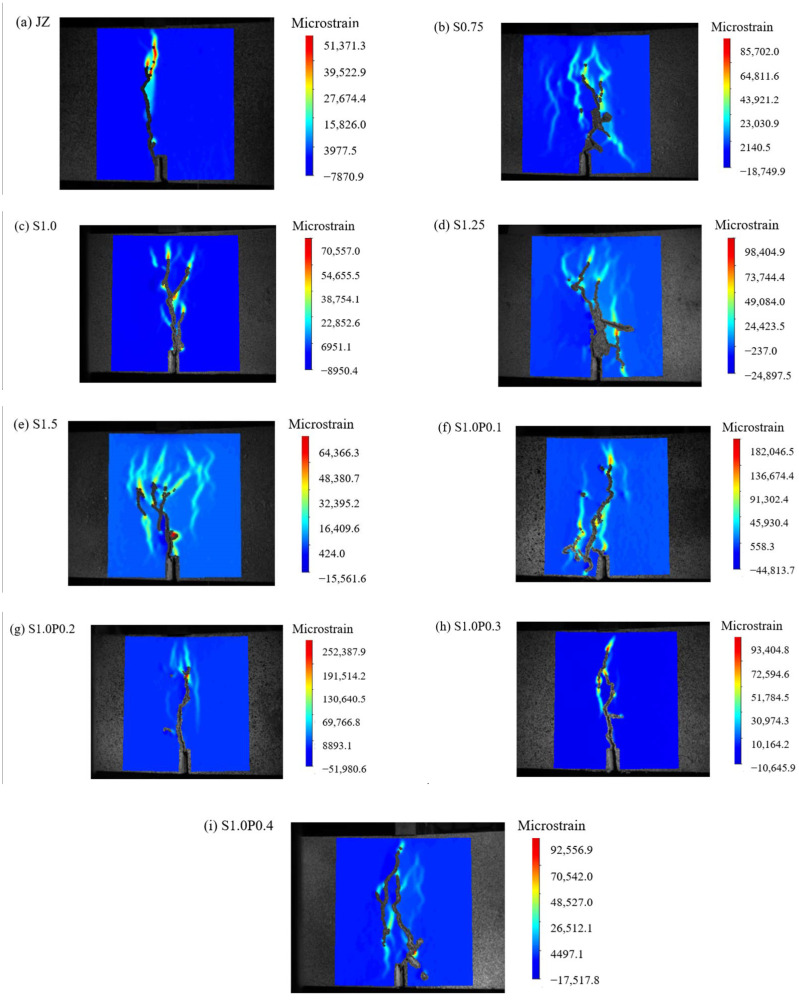
DIC measurements of concrete at *δ* of 3 mm.

When the δ is 3 mm, the DIC measurements for plain concrete, SFRC, and S-PVA HFRC are shown in Figure 9.

As can be seen in Figure 9:

(1) Prior to reaching a δ of 3 mm, the reference concrete already exhibited a penetrating crack.
(2) The addition of steel fibers delayed the development of cracks in the concrete specimens. It also increased the maximum micro-strain at the crack tip. However, the presence of steel fibers resulted in multiple cracks, as they altered the crack propagation direction. This led to an increase in the ductility of the concrete specimens.
(3) In comparison to SFRC, S-PVA HFRC showed a reduction in the number and area of cracks. The addition of PVA fibers in addition to steel fibers increased the maximum micro-strain in the SFRC. The PVA fibers limited the development of micro-cracks in the SFRC, enhanced the resistance to steel fiber pull-out, and further decreased the crack area in the concrete.

### 3.2. Concrete F-CMOD Curves

#### 3.2.1. SFRC F-CMOD Curves

**Figure 10:**
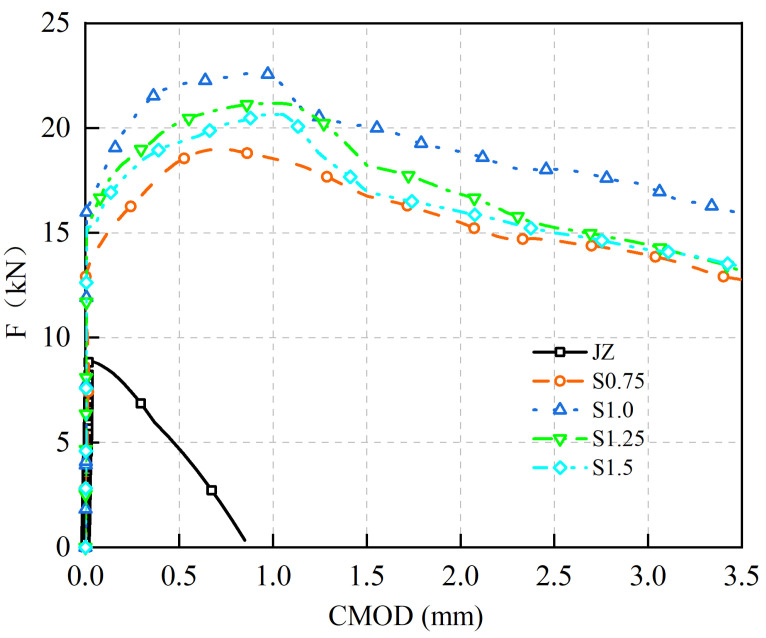
F-CMOD curves of SFRC.

F-CMOD curves of concrete specimens with steel fiber $V_{f}$ of 0% to 1.5% are shown in Figure 10.

Based on Figure 10:

(1) F increases approximately linearly with CMOD until the concrete cracks.
(2) The F-CMOD curve of the reference concrete specimen without steel fibers rapidly decreases after reaching its peak *F* as CMOD increases.
(3) Adding steel fibers not only enhances the $f_{u}$ of concrete but also leads to a smoother descending segment and a fuller curve of the F-CMOD relationship. As the $V_{f}$ of steel fibers increases, the $f_{u}$ of concrete and the enveloping area of the F-CMOD curve initially increase and then decrease. The maximum values are achieved at a steel fiber dosage of 1.0%, with an $f_{u}$ of 7.25 MPa. Compared to the reference concrete without steel fibers, the $f_{u}$ increased from 2.86 MPa to 7.25 MPa, representing a 153% improvement.

These results indicate that steel fibers effectively bridge macro-cracks in the concrete, restrict crack propagation, enhance the ductility of the concrete material, and improve the $f_{u}$. However, it is important to note that more steel fibers are not necessarily better. When the $V_{f}$ exceeds 1.0%, the clustering of the steel fibers may occur within the concrete, leading to a reduction in the strength and ductility of the concrete.

Comparative analysis of previous research results shows that for steel fiber to improve the $f_{u}$ of concrete, Bai has similar findings. It was found that as the content of steel fiber increases from 20 kg/m^3^ to 110 kg/m^3^, the $f_{u}$ also increases, but the optimal $V_{f}$ of steel fiber for improving concrete $f_{u}$ has not been found yet. Chen et al. encountered a similar problem [24]. In general, the $f_{u}$ of the panel concrete is the highest when the $V_{f}$ of steel fiber is 1.0%.

#### 3.2.2. S-PVA HFRC F-CMOD Curve

**Figure 11:**
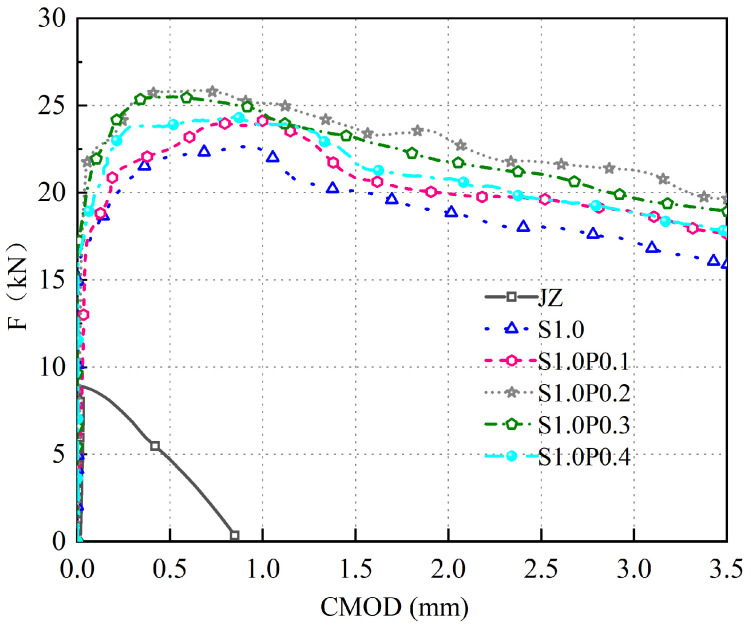
F-CMOD curve of S-PVA HFRC.

The F-CMOD curves for concrete specimens with varying volumes of S-PVA HF are shown in Figure 11.

As can be seen in Figure 11:

(1) The S-PVA HFRC exhibits a linear increase in F with the increase in CMOD during the early cracking stage.
(2) PVA fibers further enhance the $f_{u}$ and the enveloping area of the F-CMOD curve in SFRC. When the PVA fiber $V_{f}$ increases from 0.1% to 0.4%, the $f_{u}$ of S-PVA HFRC initially increases and then decreases. At 1.0% $V_{f}$ of steel fibers and 0.2% $V_{f}$ of PVA fibers, the S-PVA HFRC exhibits the highest $f_{u}$. Compared to concrete with 1.0% steel fiber $V_{f}$, the $f_{u}$ is increased from 7.25 MPa to 8.28 MPa, representing a 14.2% improvement.
(3) These results indicate that S-PVA HF can improve the $f_{u}$ of SFRC. The inclusion of PVA fibers increases the compactness of the matrix and enhances the pull-out resistance of the steel fibers within the cement matrix. The hybrid effect of S-PVA HF significantly improves the $f_{u}$ of SFRC. However, excessive PVA fiber content will lead to the aggregation of steel fibers and PVA fibers, resulting in reduced $f_{u}$ and ductility of S-PVA HFRC.

Comparative analysis of previous research results shows that Liu [42] found a similar finding. Namely, that as the PVA fiber $V_{f}$ continues to increase, the deformation ability of concrete will decrease. However, the reason for the difference in the optimal $V_{f}$ found in this article may be due to the different sizes of coarse aggregate and different concrete. In general, when 1.0% steel fiber and 0.2% PVA fiber are mixed, the $f_{u}$ of panel concrete is the highest.

### 3.3. Concrete Bending Strength

#### 3.3.1. SFRC

**Figure 12:**
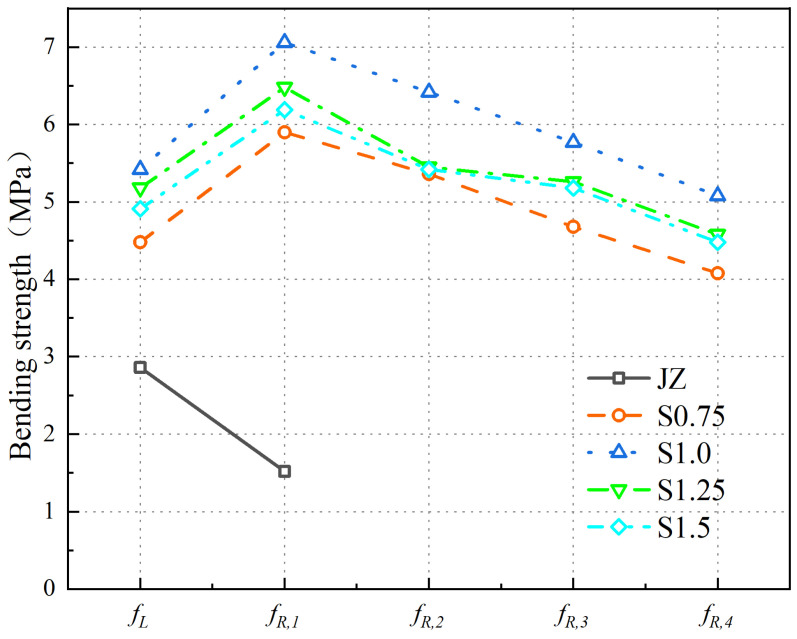
Variation Patterns of *f*_L_ and *f*_R,J_ in SFRC.

When the $V_{f}$ of steel fibers increases from 0% to 1.5%, the variation patterns of the $f_{L}$ and $f_{R,j}$ of the concrete are shown in Figure 12.

From Figure 12, the following can be observed:

(1) With an increase in steel fiber $V_{f}$, $f_{L}$ initially increases and then decreases. The $f_{L}$ of SFRC is maximum at a steel fiber $V_{f}$ of 1.0%. Compared to the plain concrete, the $f_{L}$ value increases from 2.86 MPa to 5.64 MPa, resulting in a 97.2% improvement.
(2) The $f_{R,j}$ of the reference concrete is only $f_{R,1}$, with a strength value of 1.52 MPa. There are no $f_{R,2}$, $f_{R,3}$, or $f_{R,4}$ values for the reference concrete. When the steel fiber $V_{f}$ ranges increase to 1.5%, the values of $f_{R,1}$, $f_{R,2}$, $f_{R,3}$, and $f_{R,4}$ initially increase and then decrease, with the maximum values achieved at a 1.0% steel fiber $V_{f}$.

The results indicate the following:

(1) An appropriate amount of steel fibers improves the compactness of the concrete matrix and enhances its strength, thereby increasing the $f_{L}$ of the concrete.
(2) Due to the brittle nature of the plain concrete, after reaching the $f_{u}$, F rapidly decreases with increasing CMOD, resulting in the reference concrete having only $f_{R,1}$ as its $f_{R,j}$. In the case of SFRC, as the steel fiber $V_{f}$ increases to 1.0%, more steel fibers participate in crack arrest, leading to an increase in the $f_{R,j}$ of the concrete. However, when the steel fiber $V_{f}$ exceeds 1.0%, an excessive amount of steel fibers can cluster together and fail to effectively contribute to crack arrest, resulting in a reduction in the $f_{R,j}$ of the concrete.

Comparative analysis of previous research results shows the following:

(1) Some scholars found that for C40 concrete specimens, as the 3D steel fiber content increased to 110 kg/m^3^, $f_{L}$ also increased, reaching its maximum at 70 kg/m^3^. Relevant scholars found that as the steel fiber $V_{f}$ increased to 1.5%, $f_{L}$ showed an increased trend and did not find the optimal steel fiber $V_{f}$ to increase $f_{L}$. The reason for the difference in this study may be that concrete is different, and the optimal range of steel fiber $V_{f}$ that affects the $f_{L}$ of concrete is different.
(2) For steel fiber to improve the $f_{R,j}$ of concrete, it was found that as the steel fiber content increased to 110 kg/m^3^, the increase in $f_{R,3}$ and $f_{R,4}$ of steel fiber concrete was also greater than $f_{R,1}$ and $f_{R,2}$, but they did not find the best $V_{f}$ to improve the $f_{R,j}$ of concrete.

In summary, the $f_{L}$ and $f_{R,j}$ of the panel concrete are maximized when the steel fiber $V_{f}$ is 1.0%.

#### 3.3.2. S-PVA HFRC

**Figure 13:**
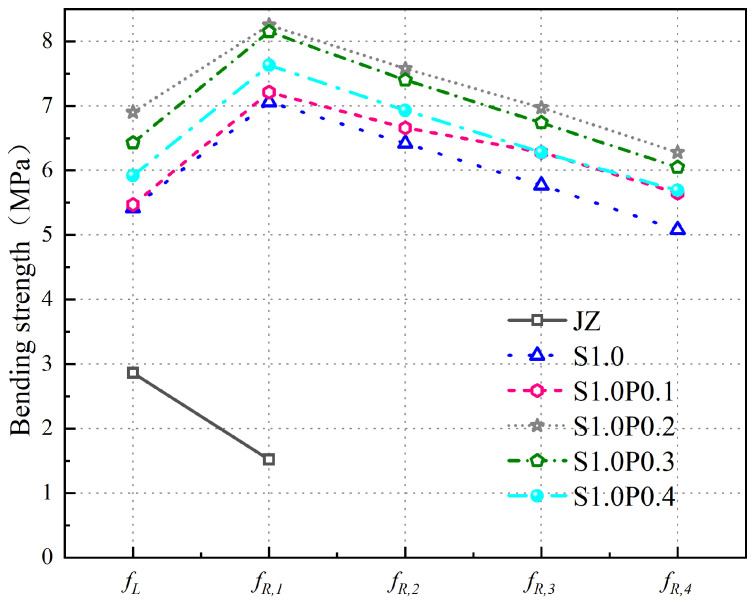
Variation of *f*_L_ and *f*_R,J_ in S-PVA HFRC.

The $f_{L}$ and $f_{R,j}$ variations of concrete specimens with varying volumes of S-PVA HF are shown in Figure 13.

From Figure 13, the following can be concluded:

(1) PVA fibers further increase the $f_{L}$ of SFRC. Compared with the $f_{L}$ of S1.0, the $f_{L}$ of S1.0P0.1, S1.0P0.2, S1.0P0.3, and S1.0P0.4 increased to 5.67 MPa, 6.90 MPa, 6.43 MPa, and 5.92 MPa, increasing by 0.5%, 22.3%, 14.0%, and 5.0%. The degree of improvement first increased and then decreased.
(2) The $f_{R,1}$ of S-PVA HFRC is greater than $f_{R,2}$, $f_{R,3}$, and $f_{R,4}$.
(3) With the increase in PVA fiber $V_{f}$, the $f_{R,j}$ of S-PVA HFRC first increases and then decreases. The $f_{R,j}$ of S1.0P0.2 is the largest. Compared with the $f_{R,j}$ of S1.0, the increase in $f_{R,1}$, $f_{R,2}$, $f_{R,3}$, and $f_{R,4}$ is higher.

The results indicate the following:

(1) The $f_{L}$ is mainly influenced by the strength of the concrete. Before reaching the proportional ultimate load, cracks first appear at the crack tip of the prefabricated incision of the concrete. Due to the reduction in the porosity of the concrete and the improvement in the density of the concrete matrix by the PVA fibers in the S-PVA HF, the generation and development of microcracks are restricted at the crack tip, which increases the $f_{L}$ of the concrete. However, as the PVA fiber $V_{f}$ is too high, the phenomenon of agglomeration between steel fibers and PVA fibers reduces the workability of concrete, thereby affecting the strength of the concrete matrix and reducing the $f_{L}$ of the concrete.
(2) The $f_{R,1}$, $f_{R,2}$, $f_{R,3}$, and $f_{R,4}$ of S-PVA HFRC, compared with the $f_{R,j}$ of concrete with 1.0% steel fiber content, increases because the multi-stage crack resistance of S-PVA HF helps to improve the load of concrete after the $f_{u}$, and improve the ductility and toughness of concrete. At the same time, PVA fiber increases the pulling resistance of steel fiber while strengthening the density of the concrete matrix. The hybrid effect of steel fibers and PVA fibers further increases energy dissipation; therefore, especially in the $f_{R,3}$, and $f_{R,4}$ stages, the degree of improvement is significantly higher than $f_{R,1}$ and $f_{R,2}$.
(3) At the same time, it is found that steel fiber has a greater effect on the $f_{L}$ and $f_{R,j}$ of concrete than PVA fiber. Compared with the benchmark concrete, the $f_{L}$ of concrete with 1.0% steel fiber increased by 2.56 MPa, $f_{R,1}$ increased by 5.54 MPa, the *f*_L_ of concrete with 1.0% steel fiber and 0.2% PVA fiber increased by 0.98 MPa, and the $f_{R,1}$ increased by 1.19 MPa, compared with concrete with 1.0% steel fiber. In terms of $f_{R,j}$, steel fibers have a more significant lifting effect on concrete than PVA fibers, but PVA fibers also play a crucial role.

Overall, S-PVA HF will further improve the resistance of SFRC.

### 3.4. Microstructure Analysis of Concrete

#### 3.4.1. SFRC

**Figure 14:**
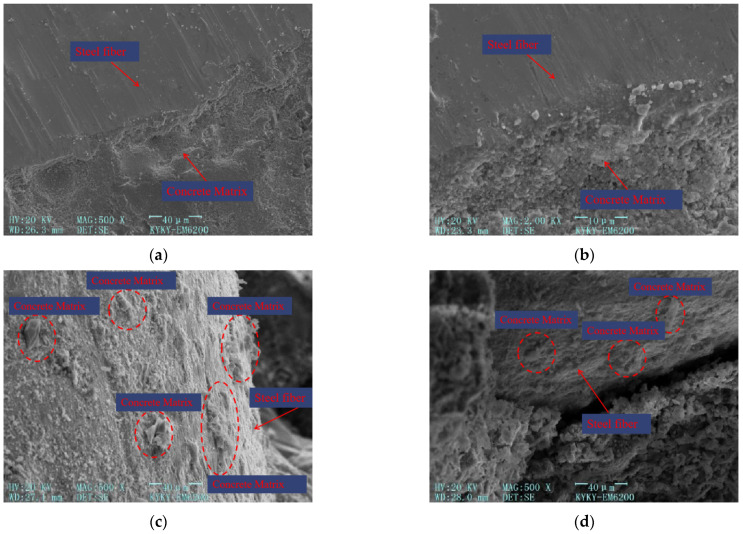
Microscopic structure image of steel fibers. (**a**) Microscopic structure image of steel fibers at 500× magnification. (**b**) Microscopic structure image of steel fibers at 2000× magnification. (**c**) Microscopic structure image of the surface of pulled-out steel fibers. (**d**) Microscopic structure image of the pulled-out steel fibers and the concrete matrix.

The microstructure of steel fibers within concrete is shown in Figure 14a,b, which depict the microstructure of the steel fiber–concrete matrix interface at 500× and 2000× magnification, respectively. Figure 14c,d represent the microstructure of pulled-out steel fibers at 500× magnification.

From Figure 14, the following can be concluded:

(1) As depicted in Figure 14a,b, the inclusion of steel fibers in the cement matrix results in a robust bond that restricts the development of macroscopic cracks. This can be attributed to the bridging effect provided by the steel fibers. Additionally, as the steel fibers unhook within the concrete, two forces come into play to further impede crack growth: the friction force between the steel fibers and the concrete, and the anchoring force exerted by the end hooks of the steel fibers embedded in the concrete. These forces work together to enhance the overall toughness and crack resistance of the concrete.
(2) In Figure 14c,d, it can be observed that when the steel fiber is pulled out, a small amount of cement-based material remains attached to its surface. This indicates that the bonding between the steel fiber and the cement-based material is limited. The relatively smooth surface of the steel fiber allows for a tight bond with the cement matrix, but the bonding force is not extensive.

The primary function of adding steel fibers is to restrict the development of macroscopic cracks, alter the direction of such cracks, bridge existing cracks, and impede crack propagation. This helps prevent internal stress concentration within the concrete, ensures a more uniform distribution of stress, and enhances its ductility.

#### 3.4.2. S-PVA HFRC

**Figure 15:**
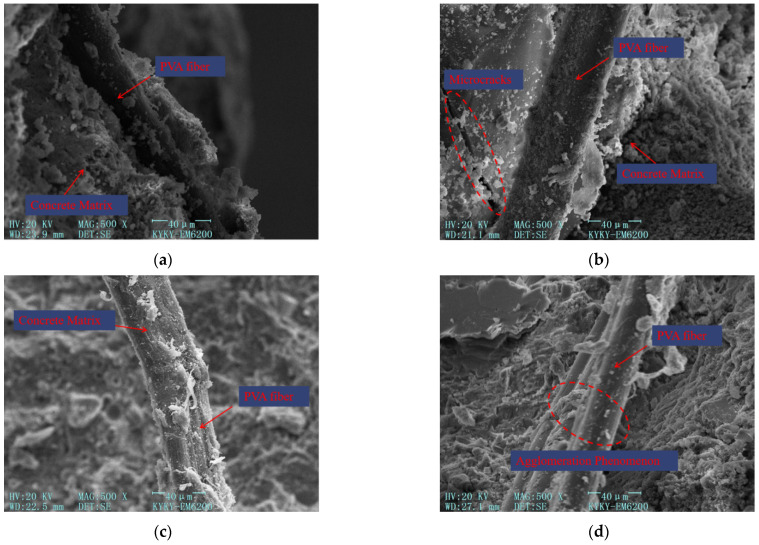
Microscopic structure image of PVA fibers within the concrete matrix at 500× magnification. (**a**) Microscopic structure image of PVA fibers within the concrete matrix. (**b**) Microscopic structure image of PVA fibers restricting microcracks. (**c**) Microscopic structure image of the surface of PVA fibers. (**d**) Microscopic structure image of clustered PVA fibers.

The microstructure of PVA fibers within the concrete material is shown in Figure 15. All the microscopic structure images of PVA fibers within the concrete matrix are at 500× magnification.

From Figure 15, the following can be concluded:

(1) Figure 15a–c illustrate the relatively uniform distribution of PVA fibers within the concrete. The surface of these fibers exhibits a rough texture without any signs of fracture. This indicates that the pulling effect of PVA fibers is optimal, as they consume more energy during the bonding and sliding processes. Consequently, the crack resistance and toughness of the concrete are improved. When stress is transmitted to the fibers through the transition zone around them, PVA fibers exhibit greater elastic deformation capacity, allowing them to effectively resist stress. In collaboration with steel fibers, PVA fibers work to suppress the formation and propagation of macroscopic cracks in the concrete.
(2) PVA fibers, as depicted in Figure 15b, pass through microcracks in the concrete. The presence of PVA fibers limits the generation and propagation of microcracks by confining the cracks on both sides of the fibers. Some microcracks may still develop and extend into areas where PVA fibers are distributed. By changing the direction of crack development and bridging the cracks, PVA fibers effectively restrict the concentrated growth of cracks. This ensures a more uniform distribution of stress throughout the entire matrix, resulting in improved strength and toughness of the cement-based materials. Furthermore, a significant amount of cement-based material can be observed adhering to the surface of PVA fibers. This strong bond between the fibers and the matrix limits the development of microcracks by tightly binding them to the matrix.
(3) But the more PVA fiber is not the better. As shown in Figure 15d, excessive PVA fiber content leads to poor dispersion of PVA fibers inside the concrete, leading to the agglomeration of PVA fibers, which increases the internal porosity of cement-based materials, and reduces concrete strength and crack resistance.

The impact of fibers of different sizes on the flexural resistance of concrete is interconnected. Each size of fiber can individually enhance the performance of cement-based materials, and the overall performance of concrete can be further improved when different sizes of fibers are combined. When steel fibers and PVA fibers are mixed, PVA fibers have the ability to fill the micropores within the cement matrix, resulting in a significant refinement of the pore structure and increased density of the microstructure. This bridging effect of PVA fibers helps to address the microcracks within the matrix. On the other hand, steel fibers primarily act as bridging elements alongside macroscopic cracks, effectively suppressing crack development. The addition of PVA fibers improves the compactness of the matrix and enhances the bonding force between steel fibers and the cement matrix. This, in turn, increases the tensile resistance of the steel fibers within the concrete. By examining the microstructure, it becomes evident that the combination of S-PVA HF plays an important role in strengthening and toughening the concrete at different scales, resulting in improved strength and flexural resistance.

## 4. Analysis of Crack Evolution Based on Fractal Theory

**Figure 16:**
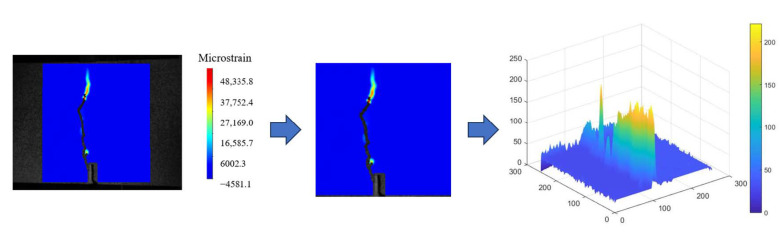
Surface grayscale set image of the reference concrete specimen.

**Figure 17:**
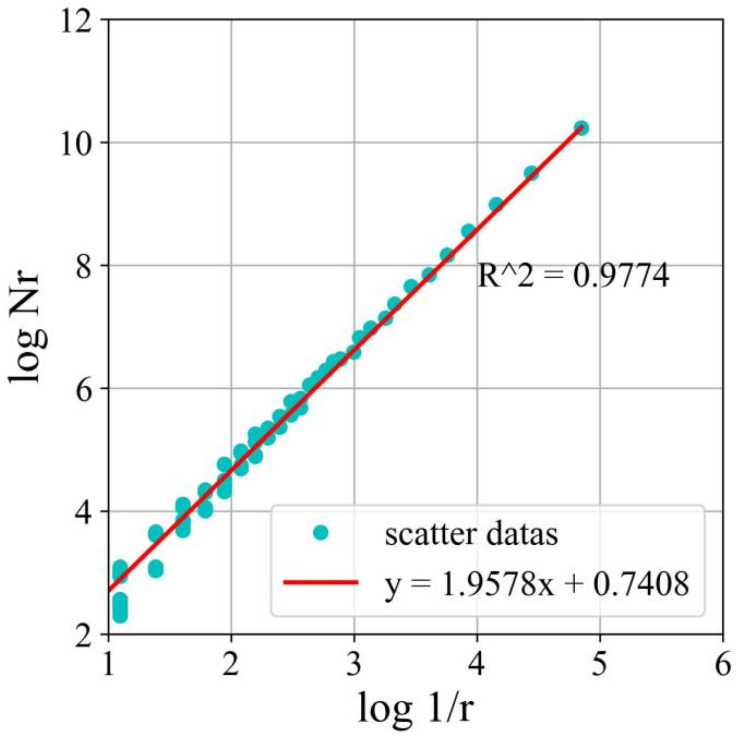
*D* of cracks in the reference concrete specimen.

The surface grayscale set of the concrete specimen’s crack development region, obtained through DIC measurement, is processed as shown in Figure 16. The *D* related to crack evolution is calculated, as shown in Figure 17.

### 4.1. SFRC

**Figure 18:**
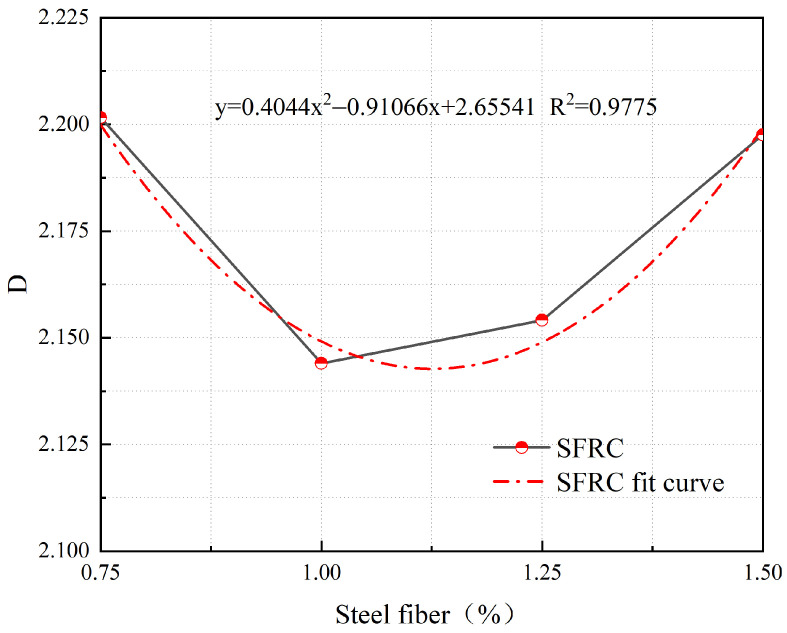
Relationship between *D* and steel fiber content at a *δ* of 3 mm.

Figure 18 shows the relationship between the *D* of cracks and the steel fiber content in concrete when the δ is 3 mm.

From Figure 18, it can be observed that at a δ of 3 mm, the *D* of cracks in SFRC exhibits a quadratic relationship with the steel fiber content. The fitted formula is as follows:

(5) $$y=0.4044x^{2}-0.91066x+2.65541$$

where *y* represents the *D* of cracks in SFRC at a δ of 3 mm, and *x* represents the $V_{f}$ of steel fibers.

No fitting analysis was performed on the reference concrete in this study as it exhibited brittle failure and had a different crack development pattern compared to SFRC and S-PVA HFRC. However, the influence of steel fiber content on crack development in concrete was quantitatively analyzed using Equation (5) with a δ of 3 mm.

Based on the results shown in Figure 18, it is evident that the *D* value of the concrete initially decreases and then increases as the steel fiber content increases. The minimum *D* value is achieved at a steel fiber content of 1.126%, indicating the optimal control effect on crack development in the concrete.

### 4.2. S-PVA HFRC

**Figure 19:**
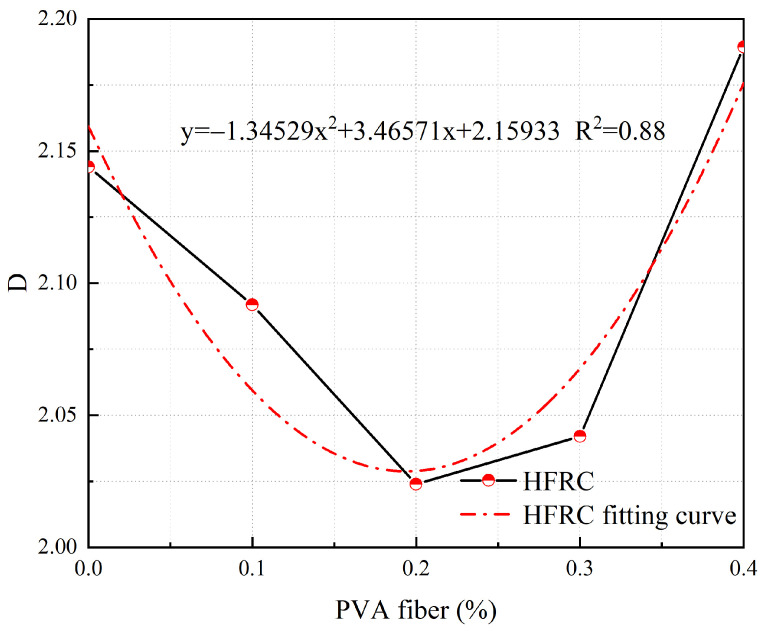
Relationship between the *D* and PVA fiber content at a *δ* of 3 mm.

At a δ of 3 mm, the relationship between the *D* of cracks and the PVA fiber content in S-PVA HFRC is shown in Figure 19.

From Figure 19, it can be observed that at a δ of 3 mm, the *D* of cracks in S-PVA HFRC exhibits a quadratic relationship with the PVA fiber content. The fitted formula is as follows:

(6) $$y=3.46571x^{2}-1.34529x+2.15933$$

where *y* represents the *D* of cracks in S-PVA HFRC at a δ of 3 mm, and *x* represents the $V_{f}$ of PVA fibers.

The quantitative analysis of crack development in S-PVA HFRC was conducted by analyzing the influence of PVA fiber content using Equation (6) with a δ of 3 mm.

From the observations in Figure 19, it is apparent that the *D* value of S-PVA HFRC initially decreases and then increases as the PVA fiber content increases. The minimum *D* value is attained at a PVA fiber content of 0.194%, indicating the optimal control effect on crack development within S-PVA HFRC.

### 4.3. Concrete Crack Evolution Analysis

**Figure 20:**
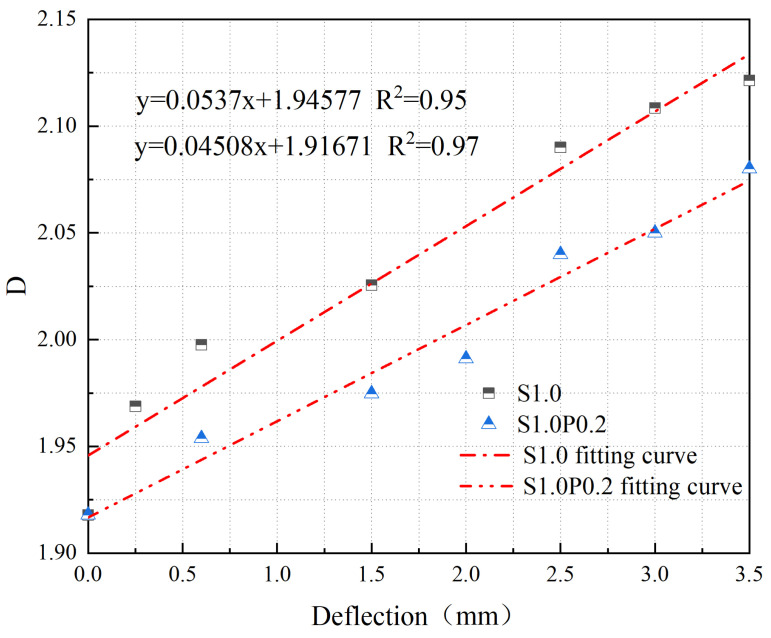
Relationship between *D* and *δ* for S1.0 and S1.0P0.2.

The relationship between the *D* of cracks and δ in S1.0 and S1.0P0.2 is shown in Figure 20.

From Figure 20, it can be observed that the *D* of cracks in SFRC and S-PVA HFRC shows a linear relationship with δ. The fitting equations are as follows:

(7) y=0.0537x+1.94577

(8) y=0.04508x+1.91671

where *y* represents the *D* of concrete cracks, and *x* represents the deflection.

According to Equations (7) and (8), when concrete undergoes ductile failure, the evolution of cracks can be quantitatively analyzed based on the *D* of cracks with increasing δ. From Figure 20, it can be seen that the *D* of cracks and the rate of crack propagation in SFRC decreases significantly with the addition of PVA fibers. The inclusion of S-PVA HF further enhances the control of cracks in concrete due to the hybrid effect between the two types of fibers.

## 5. PMFS of Concrete

Based on the analysis of experimental results and literature review, and considering the influences of steel fiber $V_{f}$ and its characteristics, PVA fiber $V_{f}$ and its characteristics, and the compressive strength of concrete on flexural strength, a PMFS is established as follows:

(9) $$f_{L}/f_{R,j}={Af_{\mathrm{cu}}}^{2/3}\left(1+\left(\left(BV_{f1}+C\right)N^{0.5}\lambda_{1}+\left(DV_{f2}+E\right)\lambda_{2}\right)\left(1+\varphi \right)\right)$$

(10) $$\lambda_{1}=V_{f1}\frac{L_{f1}}{D_{f1}}$$

(11) $$\lambda_{2}=V_{f2}\frac{L_{f2}}{D_{f2}}$$

In the formula: *L*—Fiber length (mm); *D*—Fiber diameter (mm); see abbreviations for other parameters.

Using Origin (version 2022) linear fitting is performed on the $f_{L}$ and $f_{R,j}$ of S-PVA HFRC, and the obtained values for A, B, C, D, E, and φ are shown in Table 4.

**Figure 21:**
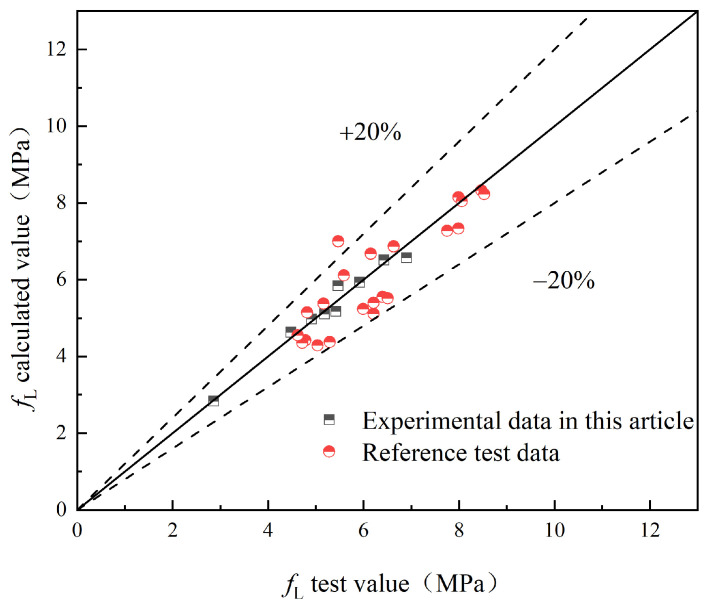
*f*_L_.

**Figure 22:**
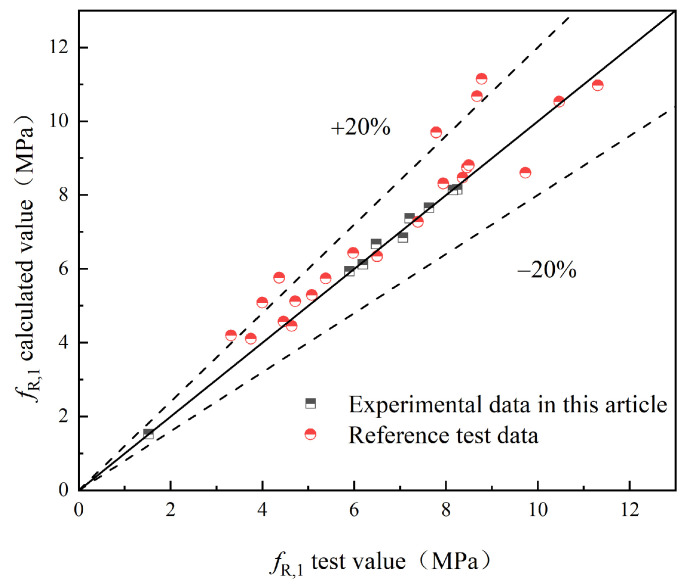
*f*_R,1_.

**Figure 23:**
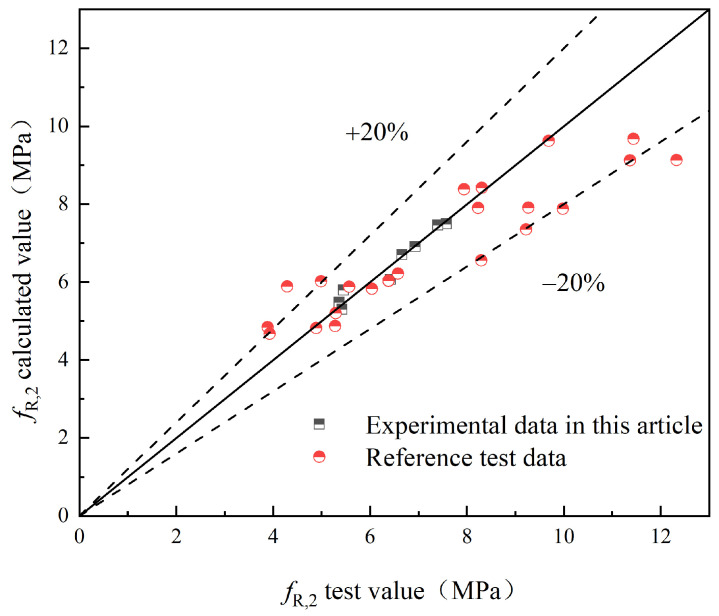
*f*_R,2_.

**Figure 24:**
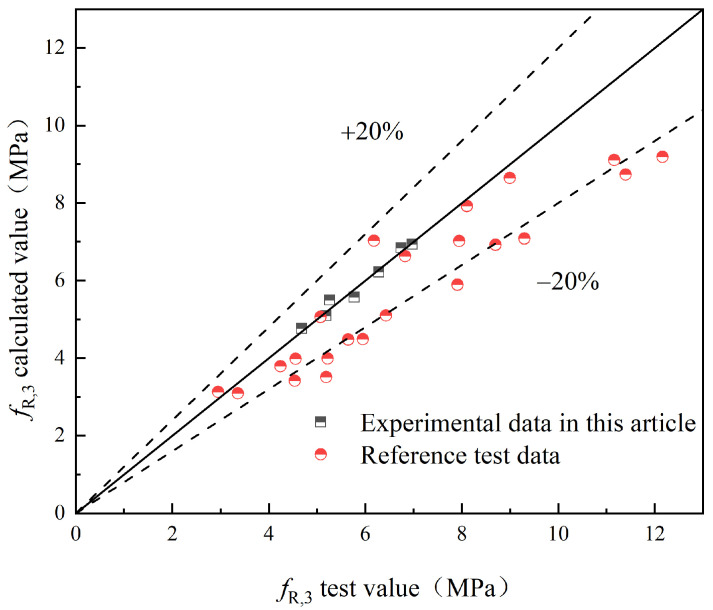
*f*_R,3_.

**Figure 25:**
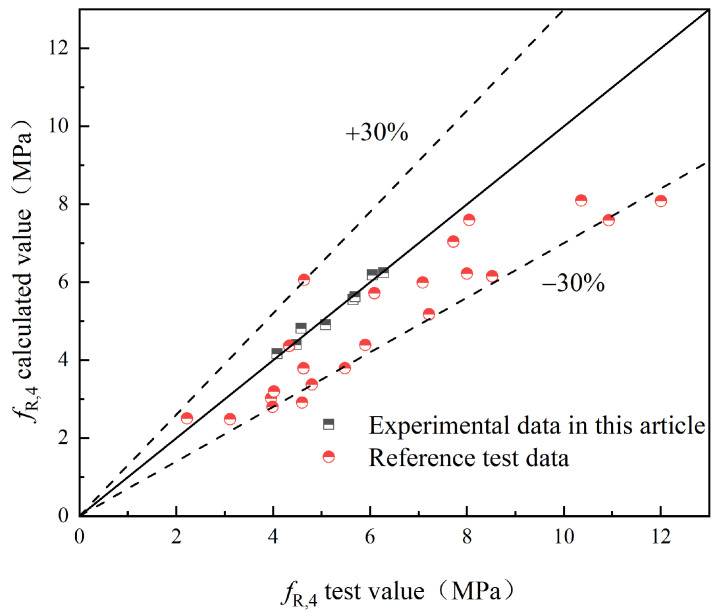
*f*_R,4_.

To validate the proposed model, this study compares the experimental values with the predicted values obtained from the model. Additionally, experimental results from relevant references [25,29] are introduced for further validation. The comparison between the experimental values ($f_{L}$ and $f_{R,j}$) and the predicted values of S-PVA HFRC, as well as SFRC from the relevant reference literature, is shown in Figure 21, Figure 22, Figure 23, Figure 24 and Figure 25.

Based on Figure 21, Figure 22, Figure 23, Figure 24 and Figure 25, it can be observed that the deviations between the calculated and experimental values of $f_{L}$, $f_{R,1}$, $f_{R,2}$, and $f_{R,3}$ are within 20%, while the deviation for $f_{R,4}$ is within 30%. These findings demonstrate the effectiveness of the proposed model in predicting the $f_{L}$ and $f_{R,j}$ of S-PVA HFRC with a maximum deviation of 30%. Therefore, the model shows a reliable capability in predicting the flexural strength of HFRC and SFRC.

## 6. Conclusions

This study focused on investigating the flexural performance of S-PVA HFRC and revealing its underlying mechanisms through three-point bending tests and SEM observations. The evolution of concrete cracks was observed using DIC, and fractal theory was employed to quantitatively analyze crack development patterns. Additionally, a flexural strength prediction model was established. The main conclusions drawn from this study are as follows:

1. The addition of S-PVA HF significantly enhances the $f_{L}$ and $f_{R,j}$ of the concrete. Comparison and analysis with the results of current studies show that S-PVA HF has a better effect on the flexural properties of concrete than steel or PVA alone, with a maximum increase in flexural strength of 22.3%.
2. SEM observations revealed that the S-PVA HF effectively controlled crack development at different scales, thereby enhancing the flexural performance of the concrete.
3. The S-PVA HF exhibited a significant improvement in crack control, resulting in reduced crack area and propagation rate.
4. A flexural strength prediction model for HFRC was developed, considering the influence of hybrid fibers on the concrete’s flexural strength. The reliability of the model was verified through experimental data.
5. For panel concrete, the greatest enhancement in flexural performance was achieved when incorporating 1.0% steel fibers and 0.2% PVA fibers.

These findings contribute to a better understanding of the flexural behavior of S-PVA HFRC and provide insights for its practical applications in construction.

## Figures and Tables

**Table 1:** Basic Properties of PVA Fibers.

Name	Density (g/cm^3^)	Diameter (mm)	Length (mm)	Elastic Modulus (GPa)	Tensile Strength (MPa)	Elongation (%)
RECS-15-12	1.3	0.031	12	41	1650	6

**Table 2:** Material Consumption of 1 m^3^ Panel Concrete (Unit: kg).

Test Piece Number	Water/Binder Ratio	Sand Rate	The Quantity of Materials Used for 1 m^3^ of Concrete (Unit: kg/m^3^)								
			Water	Cement	Fly Ash	Sand	Small Stones	Large Stones	Steel Fiber	PVA Fiber	Water Reducing Agent
JZ	0.4	0.35	160	320	80	644	657.8	538.2	0	0	0.40%
S0.75	0.4	0.35	160	320	80	623.42	636.78	521	58.8	0	0.40%
S1.0	0.4	0.35	160	320	80	616.53	629.74	515.24	78.5	0	0.40%
S1.25	0.4	0.35	160	320	80	609.67	622.73	509.51	98.1	0	0.40%
S1.5	0.4	0.35	160	320	80	602.79	615.71	503.76	117.75	0	0.40%
S1.0P0.1	0.4	0.35	160	320	80	616.07	629.28	514.86	78.5	1.3	0.40%
S1.0P0.2	0.4	0.35	160	320	80	615.62	628.81	514.48	78.5	2.6	0.40%
S1.0P0.3	0.4	0.35	160	320	80	615.16	628.35	514.1	78.5	3.9	0.40%
S1.0P0.4	0.4	0.35	160	320	80	614.71	627.88	513.72	78.5	5.2	0.40%

Note: JZ represents the base concrete, S represents steel fibers, P represents PVA fibers, and SmPn represents concrete with a $V_{f}$ of m% for steel fibers and n% for PVA fibers.

**Table 3:** Compressive Strength of Concrete.

Test Piece Number	JZ	S0.75	S1.0	S1.25	S1.5	S1.0P0.1	S1.0P0.2	S1.0P0.3	S1.0P0.4
Compressive strength (MPa)	43.7	44.17	48.66	46.64	45.64	49.5	50.7	50.64	50.04

**Table 4:** Fitting values of the flexural strength prediction model for S-PVA HFRC.

Flexural Strength	A	B	C	D	E	φ	R^2^
$f_{L}$	0.22923	−17.32713	0.43951	−52.38159	0.25589	2.22962	0.95
$f_{R,1}$	0.12327	−29.83773	0.68098	−24.41836	0.12618	9.28956	0.99
$f_{R,2}$	0.26386	−24.83106	0.51745	−43.15311	0.21698	2.31504	0.93
$f_{R,3}$	0.10383	−25.44404	0.60194	−35.56586	0.16902	9.79513	0.97
$f_{R,4}$	0.06118	−28.7121	0.66761	−38.50862	0.18742	15.3302	0.97

## Data Availability

The raw data supporting the conclusions of this article will be made available by the authors on request.

## Abbreviations

S-PVA HF	steel–PVA hybrid fibers
DIC	Digital Image Correlation
SEM	Scanning Electron Microscope
SFRC	steel fiber-reinforced concrete
HFRC	hybrid fiber-reinforced concrete
PVA	Polyvinyl alcohol
S-PVA HFRC	steel–PVA hybrid fiber concrete
CMOD	Crack Mouth Opening Displacement
δ	Deflection (mm)
F	Load (kN)
$f_{u}$	Ultimate flexural strength (MPa)
$F_{u}$	Ultimate flexural load (N)
$f_{L}$	Proportional ultimate strength (MPa)
$F_{L}$	Proportional ultimate load (N)
$f_{R,j}$	Residual flexural strength (MPa) when CMOD = CMOD_j_ (j = 1, 2, 3, 4)
$F_{j}$	Load value (N) corresponding to CMOD = CMOD_j_ (j = 1, 2, 3, 4)
*B*	Specimen cross-section width (mm)
$h_{\mathrm{sp}}$	Effective height of the specimen cross-section (mm)
$F_{\mathrm{cu}}$	Compressive strength of cubic specimens (MPa)
*N*	Number of end hooks of steel fibers
λ	Fiber characteristic values
$V_{f}$	Volume fraction
φ	Hybridization effect coefficient
PMFS	predictive model for flexural strength
*D*	fractal dimension
